# Hypocitraturia as a biomarker of renal tubular acidosis in patients with Sjögren’s disease

**DOI:** 10.1186/s42358-024-00387-7

**Published:** 2024-06-03

**Authors:** Rafael Coradin, Maria Lúcia Lemos Lopes, João Carlos Goldani, Pedro Enrico Ventura, Elizete Keitel

**Affiliations:** Federal University of Health Sciences of Porto Alegre, Porto Alegre, Brazil

**Keywords:** Sjögren’s disease, Renal involvement, Distal renal tubular acidosis, Tubulointerstitial nephritis, Biomarker, Hypocitraturia

## Abstract

**Introduction:**

Sjögren’s disease (SD) is an immune-mediated chronic inflammatory disease that affects epithelial tissues, mainly salivary and lacrimal glands. It also presents extraglandular manifestations. The main renal manifestation is tubulointerstitial nephritis (TIN), which can manifest as renal tubular acidosis (RTA). Urinary citrate may be a biomarker of RTA in these patients. The objective of this study was to evaluate whether hypocitraturia is a predictive biomarker of RTA in a sample of patients with SD in a tertiary hospital in southern Brazil.

**Methods:**

All patients with SD who met the inclusion criteria and who participated in the rheumatology outpatient clinic of the Irmandade Santa Casa de Misericórdia de Porto Alegre were included. Demographic, SD, serological and urinary data were obtained. RTA was considered in those patients who persistently presented urinary pH above 5.5 and serum pH below 7.35. Patients who persistently had urinary pH above 5.5 underwent a urinary acidification test with furosemide and fludrocortisone. These patients received 1 mg of fludrocortisone and 40 mg of furosemide and had their urine samples tested 2, 4 and 6 h after taking the medications. The test was stopped at any urine sample with pH 5.5 or less. The variables were expressed as mean and standard deviation or interquartile range. The association between hypocitraturia and RTA was assessed using the chi-square.

**Results:**

Forty-two patients were included, 95.2% female with a median age of 61.73 years. The prevalence of complete distal RTA was 4.88%. Twenty-eight patients underwent urine acidification testing. Five patients had hypocitraturia, and two of them had complete distal RTA. The association between hypocitraturia and RTA was statistically significant (*p* < 0.012), with a sensitivity of 100%, specificity of 91.2% and accuracy of 91.7%. The negative predictive value was 100%. The global renal assessment of the population demonstrated two patients with RTA, one patient with decreased renal function and six patients with proteinuria greater than 0.5 g/24 h.

**Conclusion:**

The prevalence of RTA in the studied population was 4.88%. Hypocitraturia had high sensitivity and accuracy for the diagnosis of RTA.

## Introduction

Sjögren’s disease (SD), former known as Sjögren’s syndrome (SS) [1], is a chronic inflammatory disease that affects the epithelium, especially the exocrine glands, with a predilection for the lacrimal and salivary glands. It has an insidious and progressive evolution, characterized histologically by a lymphoplasmacytic infiltrate.

This disease also presents extraglandular manifestations such as joint, pulmonary, central, and peripheral nervous system involvement, as well as renal involvement [2–5].

The prevalence in the general population diverges in the literature, depending on the population studied. It varies from 3.3% in the United Kingdom [6], 0.13–0.42% in the United States [7], 16% in Spain [8] and 0.17% in Brazil [9].

The clinical presentation of tubulointerstitial nephritis (TIN) in SD is varied. Affected patients may present signs of renal tubular acidosis (RTA), generally type 1, with RTA being complete or incomplete, nephrogenic diabetes insipidus, nephrolithiasis or nephrocalcinosis, or muscle weakness [2, 4]. Its prevalence also varies according to the population studied.

The evaluation of renal involvement in SD is performed through the analysis of several biochemical parameters in the patient’s serum and urine. It is essential to perform blood gas analysis and serum levels to assess the presence of metabolic acidosis, serum creatinine and electrolytes, especially potassium. In the analysis of the patient’s urine, the urinary pH (potential of hydrogen), electrolytes, creatinine and presence of proteins should be evaluated. All patients should also undergo radiological evaluation of the kidneys. Patients with a persistent urinary pH above 5.5 should be evaluated for RTA, which can be defined as complete, when the blood pH is less than 7.35, or incomplete, when the blood pH is greater than or equal to 7.35 and there is inability to acidify the urine when evaluated by some urine acidification test [10–16].

Some TIN biomarkers have been evaluated to obtain an early marker of renal impairment in patients with SD, with emphasis on b-2 microglobulin, a1 microglobulin, and urinary citrate; however, there is a lack of robust scientific evidence to prove its efficacy as predictors of nephritis [17–21]. A correlation was also observed between renal involvement with anti-La (SSB) positivity [22] or even increased serum IgG and renal involvement [23].

The aims of the present study were to analyze the accuracy of low urinary citrate in the diagnosis of RTA and to evaluate renal involvement and the sociodemographic profile of a population of patients with Sjögren’s disease.

## Materials and methods

This is a cross-sectional, prospective study evaluating renal involvement in a population with SD not associated with other immune-mediated rheumatological diseases treated at a rheumatology outpatient clinic at a tertiary hospital in southern Brazil.

The sample of the study population was determined by convenience, with all patients diagnosed with SD who agreed to participate in the study being included. The period of inclusion in the study was from March 2021 to February 2022.

The inclusion criteria in the study were as follows: age over 18 years old, Sjögren’s disease not associated with other immune-mediated rheumatological diseases according to the classification criteria of the American College of Rheumatology—European Alliance of Associations for Rheumatology (ACR-EULAR) Classification Criteria for Primary Sjögren Syndrome of 2016 [24] and written informed consent to participate in the study. The study exclusion criteria were the presence of other immune-mediated rheumatological diseases, allergies or medical contraindications to the use of fludrocortisone and/or furosemide and participants who withdrew their written informed consent to participate in the study.

Demographic data were obtained (age, gender, ethnicity, education, occupation and family income), data related to SD (diagnosis delay time, defined as the difference between the date of diagnosis and the date of onset of clinical symptoms), immunological data of the disease (anti-nuclear factor—ANA, rheumatoid factor, anti-Ro (SSA) anti-La (SSB), complement fractions), and serum laboratory data (hemogram, creatinine, urea, sodium, potassium, chloride, alkaline reserve, blood gas measurements, serologies for hepatitis B and C, for acquired immunodeficiency virus—HIV). The urinary data were collected from a sample for urinalysis and a 24-h urine sample for measurement of protein, creatinine, and citrate. Abdominal X-ray data were collected from medical records.

The diagnosis of RTA was considered in all participants who had metabolic acidosis (blood pH less than 7.35) with a normal serum anion gap, positive urinary anion gap and urinary pH greater than 5.5 [10]. This study only evaluated the presence of type 1 RTA.

To determine complete or incomplete RTA, the last three urine tests recorded in the patient’s medical record were analyzed. Patients who already had a urinary pH below 5.5 were exempt from undergoing the urine acidification test. Those who presented urinary pH above 5.5 in these three records, regardless of blood pH and in the absence of urinary infection, were invited to perform the urine acidification test with fludrocortisone and furosemide [25]. This test consisted of a baseline urinalysis and serum alkaline reserve and then oral administration of 1 mg of fludrocortisone and 40 mg of furosemide. Urine samples were obtained after 2 h and submitted to a pH test with a dipstick. Patients who had acidified urine were referred for a new serum collection for alkaline reserve, and the test ended. Patients who did not acidify their urine within 2 h underwent a new urine sample collection and alkaline reserve 4 and 6 h after medication administration. As soon as the urine sample was acidic, the test was finished. Patients who did not acidify their urine 6 h after taking the medication also had their test ended.

The diagnosis of complete distal RTA was considered in patients who had persistent urinary pH above 5.5 and serum pH below 7.35. Incomplete distal RTA was considered in those patients with urinary pH persistently above 5.5, serum pH greater than or equal to 7.35 and who did not acidify the urine during the test.

Low urinary citrate was considered in those patients who presented citraturia lower than 320 mg/24 h (lower limit of the referenced laboratory test).

Statistical analysis was performed using SPSS 20.0 software. Normal distribution was analyzed using the Kolmogorov‒Smirnov test. Continuous variables with a normal distribution were expressed as the mean and standard deviation, and those without a normal distribution were expressed as the median and interquartile range. Categorical variables are described as numbers and frequencies. To analyze the association between renal tubular acidosis and hypocitraturia, the chi-square test was performed. The accuracy of the test was calculated. The determination of the best cutoff point for citraturia was defined using the ROC curve.

All participants signed a written informed consent, and the present study was approved by the Ethics Committees of the Federal University of Health Sciences of Porto Alegre—UFCSPA (# 4.546.427) and Santa Casa de Misericordia of Porto Alegre Hospital Complex– ISCMPA (# 5.062.089).

## Results

Initially, 45 patients were included. Subsequently, three were excluded for withdrawing informed consent. Six patients’ data were incomplete, 3 due to death and 3 for not performing all the tests during the study period.

Demographic data are described in Table 1. Most participants included in the study were female, with a median age of 61.73 years (52.50–69.07), white ethnicity, low education and family income of up to 2 minimum wages.

**Table 1:** Demographic data of the study population

		Median	IQR^¥^
Age* (*n* = 42)		61.73	52.50–69.07
Sex (*n* = 42)		**N**	**%**
	Female	40	95.2
	Male	2	4.8
Ethnicity (*n* = 40)	White	32	76.2
	Brown	6	14.3
	Black	2	4.8
Education (*n* = 36)	Elementary	25	59.5
	High school	8	19.1
	College	3	7.2
Occupation (*n* = 41)	Hired	10	23.8
	Self-employed	10	23.8
	Retired	13	31.0
	Housewive	7	16.7
	Student	1	2.4
Origin (*n* = 42)	Capital and metropolitan region	32	76.2
	Countryside	10	23.8
Family Income^±^ (*n* = 35)	<1	2	4.8
	1 to 2	24	57.1
	2 to 3	3	7.1
	3 to 4	3	7.1
	>4	3	7.1

*In years

^¥^Interquartile range

^±^In minimum wages

All patients included in the study had negative serum antibodies for hepatitis B, hepatitis C and HIV.

The clinical and immunological data are shown in Table 2. The median delay in the diagnosis of the study participants was 4.5 years (1.50–9.50). Most participants were positive for ANA and anti-Ro (SSA). There were no changes in the serum levels of complement fractions (C3 and C4) in all patients included in the study. The 4 participants included in the study were anti-Ro (SSA) negative and had positive minor salivary gland biopsy (Focal Score ≥ 1) [26] for SD.

**Table 2:** Clinical and immunological data

		Median	IQR^¥^
Diagnosis delay time* (*n* = 40)		4.5	1.50–9.50
ANA (*n* = 41)		**n**	**%**
	Positive	37	88.1
Rheumathoid factor	Positive	19	45.2
ANTI-Ro (SSA) (*n* = 41)	Positive	37	90.3
ANTI-La (SSB) (*n* = 40)	Positive	14	33.3

*In years

^¥^Interquartile range

Table 3 summarizes the analysis of data from the evaluation of renal impairment. Twenty-eight participants underwent the urine acidification test with fludrocortisone and furosemide.

**Table 3:** Renal involvement evaluation data

		Mean	Standard deviation (SD)
Serum potassium (mEq/L) (*n* = 40)		4.5	± 0.60
Venous gasometry (*n* = 35)	pH	7.37	± 0.04
	HCO3	26.29	± 2.5
	Alkaline reserve	30.82	± 3.37
Urinary citrate in 24 h (mg/24 hours) (*n* = 36)		1009.77	± 594.05
Urine analysis (*n* = 40)	pH	**Median**	IQR*
		6.00	5.50–6.50
24 h Protein (g/dL) (*n* = 40)		0.18	0.09–0.31
Glomerular filtration rate (ml/min/1.73 m^2^) (*n* = 36)		101.0	94.00–109.00

*Interquartile range

Two participants had metabolic acidosis on blood gas analysis, with a normal serum anion gap and positive urinary anion gap (both did not acidify the urine) and were diagnosed with complete renal tubular acidosis. The prevalence of RTA in this population was 4.88% (2/42).

The association of hypocitraturia with RTA was statistically significant (*p* < 0.012). Hypocitraturia showed 100% sensitivity and 91.2% specificity. The accuracy of the method was 91.7%, the positive predictive value (PPV) was 40% and the negative predictive value (NPV) was 100%. The Cutoff with best sensitivity and specificity was 177.8 mg/24 h of citraturia. The sensitivity was 100% and the specificity was 99.97%, with an area under the curve for hypocitraturia of 0.985 (*p* 0.023–95% CI 0.95–1.00).

None of the participants had hypokalemia, and the median urinary pH was 6.00 (IQR 5.50–6.50). Urinary citrate had a mean of 1009.77 ± 594.05 mg/24 h.

Six patients had proteinuria greater than 0.5 g/24 h.

Of the six individuals highlighted, it is noted that only one of them had acidic urinary pH and that half of them had serum potassium closer to the lower limit of normality. One patient had high serum potassium and a glomerular filtration rate below 60 ml/min/1.73 m^2^. Patient number 7 was diagnosed with complete renal tubular acidosis.

Five subjects had low urinary citrate at 24 h (less than 320 mg/24 h). Patients 7 and 41 were those diagnosed with complete RTA. Data regarding citraturia are described in Table 4.

**Table 4:** Data related to patients with hypocitraturia

Patient *n*.	Creatinine (mg/dL)	Potássio sérico (mEq/L)	Gasometry		pH urinário	Urinary citrate (mg/24 h)	Urinary protein (g/24 h)	Urinary creatinine (g/24 h)	Glomerular Filtration Rate (ml/min/1.73 m^2^)
			pH	HCO3					
07	1.05	3.7	7.28	22.40	7.00	36.00	0.58	0.80	103
09	0.71	5.3	7.36	26.80	5.50	112.70	0.06	0.80	103
12	0.89	4.2	7.40	29.30	5.00	194.20	0.18	0.80	91
17	0.72	4.6	7.32	29.90	5.50	60.20	0.23	0.87	101
41	0.68	4.8	7.33	25.30	6.50	161.64	0.09	1.34	121

Patients 7, 17 and 41 had acidosis. Patients 7 and 41 had normal bicarbonate and elevated pCO2, presenting mixed acidosis. Patient 17 already had a urinary pH lower than 5.5, while patient 41 had a urinary pH greater than 5.5. The glomerular filtration rate of the studied population was reduced in 6/39 patients (15.79%).

The evaluation of the immunological data showed that patient 7 had positive ANA, anti-Ro (SSA) and anti-La (SSB), negative rheumatoid factor and increased gammaglobulinemia. Patient 41 had positive ANA, rheumatoid factor and anti-Ro (SSA), negative anti-La (SSB) and normal gammaglobulinemia.

There was no statistically significant association between RTA and anti-La (SSB) positivity (*p* 0.583). The same occurred when analyzing the association between any renal manifestation and anti-La (SSB) positivity (*p* 0.300).

This study did not collect serum IgG data from the included patients.

Nephrolithiasis was present in 2 of 25 patients (4.8%) in the abdominal X-ray. One of them had RTA. None had nephrocalcinosis.

## Discussion

This study found a prevalence of complete distal RTA of 4.88%. These two patients had low urinary citrate levels. A study conducted in Norway found a prevalence of 11.3% (7/62) of patients with RTA, with 6.5% (4/62) having complete distal RTA and 4.8% (3/62) having incomplete distal RTA [18]. In another study with 715 patients with SD, the prevalence of total renal involvement was 4.9% and the prevalence of TIN was 1.8% [27]. In this study, patients were not subdivided as to the possibility of complete and incomplete distal RTA, nor was citraturia evaluated.

A study carried out in Holland with 57 patients evaluated the prevalence of complete and incomplete RTA and found a prevalence of 3 (5%) patients with complete distal RTA and 14 (25%) with incomplete distal RTA [13].

The prevalence of renal involvement, especially RTA, is higher in the Asian population. A study conducted in India found 19.66% (35/178) of patients with renal involvement, 71.42% (25/35) with complete dRTA and 11.42% (4/35) with incomplete dRTA [16]. A Chinese study with 130 SD patients with renal involvement showed a 73.1% (91/130) incidence of RTA; 72.52% (66/91) of these patients had complete distal RTA, and 24.47% (25/91) had incomplete distal RTA [23]. In another Chinese study that included 573 patients with SD, the frequency of renal involvement was 33.5% (*n* = 192), 16.7% (*n* = 96) with RTA [28].

In our study, low urinary citrate was present in 12.19% (5/41) of the included patients, two of whom had complete RTA. In the literature, we found reports of patients with SD renal tubular acidosis and hypocitraturia [29–32]. Data from Norway showed low levels of urinary citrate in 25.8% (16/62) of patients with SD. All seven patients with RTA had hypocitraturia, demonstrating a sensitivity of 100%, specificity of 83.6%, a PPV of 43.8% and an NPV of 100% [18], data very similar to those found in the present study.

Our data, together with data from the literature, point to the possibility that urinary citrate can be used as an early biomarker of RTA in patients with SD. It is a method with high sensitivity and negative predictive value. In our study, the calculated accuracy for hypocitraturia was 91.7%. The absence of hypocitraturia can be considered to rule out the diagnosis of RTA, as a highly sensitive test is very useful for clinicians when the test result is negative.

The presence of nephrolithiasis in this study was 4.8%, which is lower than that observed in other studies, especially in the Asian population, where the rates of nephrolithiasis range from 8.57 to 33% [14, 16, 33, 34].

Our study chose to perform the urine acidification test with furosemide and fludrocortisone because it is easier to perform and better tolerated by patients. Similar effectiveness rates were demonstrated by comparing the acidification test between ammonium hydrochloride (gold standard) and the furosemide plus fludrocortisone test [35]. Other studies showed sensitivity ranging from 65 to 77% and specificity ranging from 68 to 85% [13, 35].

The sociodemographic characteristics of the population in this study are quite similar to other studies [10], especially a Brazilian study [9]: the majority are women, in their fifth decade of life, white and with familial low income. However, the Asian population differs slightly in the average age of involvement around the third to fourth decades of life [16, 23].

The median time delay in diagnosis of 4.5 years in our study is similar to studies with populations from developing countries [16, 20, 23].

Our study chose to classify patients with SD only by the American College of Rheumatology—European Alliance of Associations for Rheumatology (ACR-EULAR) 2016 criteria [24], while most studies classified their patients by the American—European Consensus Group (AECG) 2002 [36] and/or ACR-EULAR 2016 criteria. We chose these criteria for convenience since all patients in the outpatient clinic were already classified according to the ACR-EULAR 2016 criteria.

This study is one of the few prospective studies that evaluated the presence of RTA in patients with SD. Our methodology did not consider to perform the urine acidification test at two different times due to the technical difficulties and costs of carrying out these tests. We also emphasize that all acidification tests were carried out by a single person to minimize errors and biases in execution. All laboratory tests were standardized. The studied sample included all patients followed at the outpatient clinic who agreed to participate.

The study limitations may be the small sample size; although the disease is rare, multicenter studies are needed to obtain larger samples. The study also only included patients from a single rheumatology center, which reduces the external validity of our data. The small number of patients with RTA may be a limiting factor in the analysis of hypocitraturia, but it was present in both patients with RTA.

The protocol of this study did not predict the performance of renal biopsy for diagnosis. One of the patients had proteinuria greater than 2 g/24 h. This patient had poor control of diabetes and high blood pressure. Proteinuria, in this case, may have another etiology, and the patient was referred for evaluation of glomerulopathy.

## Conclusion

Our sample of Sjögren’s disease patients was mostly women in their fifth decade of life who were white and had familial low income. In the evaluation of renal impairment, one patient had chronic kidney failure, and six patients had proteinuria greater than 0.5 g/24 h. The prevalence of RTA was 4.88%. Hypocitraturia showed a high sensitivity for the diagnosis of RTA with an accuracy of 91.7%.

## Data Availability

The datasets used and/or analyzed during the present study are available in the supplemental material of this article.

## Abbreviations

DS: Sjögren’s Disease
SS: Sjogren’s Syndrome
TIN: Tubulointerstitial nephritis
RTA: Renal tubular acidosis
dRTA: distal renal tubular acidosis
pH: Potential of hydrogen
SSB: Sjögren Syndrome B
IgG: Immunoglobulin G
ACR: American College of Rheumatology
EULAR: European League Against Rheumatism
ANA: Anti-nuclear factor
SSA: Sjögren Syndrome A
HIV: Human Immunodeficiency Virus
RX: X-ray
SPSS: Statistical Package for Social Science
UFCSPA: Universidade Federal de Ciências da Saúde de Porto Alegre
ISCMPA: Irmandade Santa Casa de Misericórdia de Porto Alegre
IQR: Interquartile Range
HCO3: Serum bicarbonate
PPV: Positive Predictive Value
NPV: Negative Predictive Value
GFR: Glomerular filtration rate
CKD EPI: Chronic Kidney Disease Epidemiology Collaboration
pCO2: partial pressure of carbon dioxide
